# HOTAIR promotes gefitinib resistance through modification of EZH2 and silencing p16 and p21 in non-small cell lung cancer

**DOI:** 10.7150/jca.56093

**Published:** 2021-07-25

**Authors:** Weiting Li, Yongwen Li, Hongbing Zhang, Minghui Liu, Hao Gong, Yin Yuan, Ruifeng Shi, Zihe Zhang, Chao Liu, Chen Chen, Hongyu Liu, Jun Chen

**Affiliations:** 1 Department of Lung Cancer Surgery, Tianjin Medical University General Hospital, Tianjin 300052, P.R. China.; 2 Tianjin Key Laboratory of Lung Cancer Metastasis and Tumor Microenvironment, Tianjin Lung Cancer Institute, Tianjin Medical University General Hospital, Tianjin 300052, P.R. China.

**Keywords:** Lung cancer, HOTAIR, Gefitinib resistance, EZH2

## Abstract

The long non-coding RNA Hox transcript antisense intergenic RNA (HOTAIR) plays a critical role in tumorigenesis as well as drug resistance in various cancers. However, the molecular mechanism by which HOTAIR induces gefitinib resistance in non-small cell lung cancer is to date unclear. In the present study, we revealed that HOTAIR is upregulated in gefitinib-resistant lung cancer cells and over-expression of HOTAIR enhances gefitinib resistance in lung cancer cells. In addition, the overexpression of HOTAIR promotes cell cycle progression through epigenetic regulation of EZH2/H3K27. Silencing of EZH2 by either siRNA or inhibitors sensitized the lung cancer cells to gefitinib. Inhibition of EZH2 induces expression of p16 and p21, whereas levels of CDK4, cyclinD1, E2F1, and LSD1 are significantly decreased in PC-9 cells overexpressing HOTAIR. ChIP-PCR experiments indicate that HOTAIR increases H3K27me3 recruitment to the promoter of p16 and p21 in PC-9 lung cancer cells overexpressing HOTAIR. In xenograft mouse models, overexpressing HOTAIR in lung cancer tissues decreased p16 and p21 proteins. Taken together, these data suggest that HOTAIR contributes to gefitinib resistance by regulating EZH2 and p16 and p21. Targeting HOTAIR may be a novel therapeutic strategy for treating gefitinib-resistance in non-small cell lung cancer.

## Introduction

Lung cancer is one of the leading causes of cancer-related mortality globally 1 and is responsible for approximately one quarter of all cancer deaths 2. Histologically, lung cancers are usually grouped into non-small cell lung carcinoma (NSCLC) and small cell carcinoma 3,4. NSCLC accounts for approximately 85% of all lung cancers and is divided into adenocarcinoma, squamous cell carcinoma, and large cell carcinoma 5. A better understanding of the molecular mechanisms underlying NSCLC treatment is urgently needed for developing new therapeutic targets to improve the prognosis of this disease, as although tremendous progress has been made in the treatment of NSCLC, the overall five-year survival of NSCLC is still 15%.

In the past 15 years, a new class of drugs, called tyrosine kinase inhibitors (TKI), was developed for the treatment of NSCLC. These drugs improve patient survival 6 by targeting receptor tyrosine kinase (RTK) aberrations in cancers, including epidermal growth factor receptor (EGFR), BRAF, anaplastic lymphoma kinase, RET, ROS1, and MET 7,8. Gefitinib, a TKI of EGFR, is the first-line treatment for advanced NSCLC in patients harboring activating EGFR mutations. Resistance developing around 10 months after initiating treatment is presenting tremendous challenges to the clinical application of gefitinib in cancer patients 9, despite treatment of NSCLC with gefitinib being effective. Therefore, understanding the molecular mechanisms underlying gefitinib resistance is important in discovering new therapeutic strategies for NSCLC treatments.

Long non-coding RNAs (lncRNAs) are non-protein coding transcripts ranging from 200 nucleotides to about 100 kb nucleotides. LncRNAs have emerged as novel master regulators of initiation, progression, and response to therapy in a wide variety of solid tumors and hematological malignancies 10,11. Hundreds of IncRNAs associated with lung cancer have been identified through gene expression microarrays and massively parallel RNA sequencing 12-14. The lncRNA Homeobox transcript antisense intergenic RNA (HOTAIR) is upregulated in many human cancers, including breast cancer, liver cancer, ovarian cancer, gastric cancer, pancreatic cancer, glioma, and non-small cell lung cancer (NSCLC) 15. HOTAIR is highly expressed in invasive NSCLC and is located on chromosome 12q13.13, at a regulatory boundary in the HOXC cluster 16,17. Silencing HOTAIR reduces invasiveness and reverses EMT in gastric cancer cells 18. Furthermore, the downregulation of HOTAIR may sensitize tumor cells to trastuzumab in breast cancer 19. Our previous study demonstrated that HOTAIR and its two segments, HOTAIR3' and HOTAIR5', promote cell cycle progression through the restriction point during the G1-S phase by regulating the Rb-E2F1 pathway 20. In this manner, HOTAIR influences NSCLC cell proliferation, migration, and invasion. Our previous research also revealed that elevated HOTAIR expression induces resistance to gefitinib through dysregulation of the cell cycle. However, the detailed mechanism of HOTAIR-induced drug resistance remains poorly understood.

The present study was devised to elucidate the contributions of HOTAIR to gefitinib resistance in NSCLC and to explore the underlying mechanism. We investigated whether HOTAIR regulates NSCLC cell growth, proliferation, and resistance by mediating the EZH2/ H3 lysine 27 (H3K27me3) signaling pathway. The results of this study contribute to a better understanding of the molecular mechanisms of HOTAIR-induced drug resistance and provide a new perspective for NSCLC treatment.

## Materials and Methods

### Cell culture

PC9 and the gefitinib-resistant cell line, PC9/AB2, were generous gifts from Prof. Zhou of the Shanghai Pulmonary Hospital, Shanghai, China. PC9 is a gefitinib-sensitive cell line that harbors a deletion in EGFR exon 19 (E746-A750del). Gefitinib resistance in PC9/AB2 cells was established by long-term exposure of PC9 cells to gefitinib^21^.HCC827 was purchased from the American Type Culture Collection (ATCC; Manassas, VA, USA).The use of the cell lines was approved by the ethics committee of the Tianjin Medical University General Hospital. All cell lines were cultured in RPMI 1640 medium supplemented with 10% fetal bovine serum (FBS; Life Technologies, Carlsbad, CA). DZNeP, 3-deazaneplanocin A, EED226, and Gefitinib (N-(3-chloro-4-fluorophenyl) -7-met hoxy -6-[3-(morpholin-4-yl) propoxy] quinazolin-4-amine) were purchased from Selleck Chemicals, LLC (Houston, USA). All drugs were dissolved in DMSO and PC9/AB2 cells were exposed to gefitinib concentrations of 10-25 nmol/L.

### Transient transfection and lentiviral infection

The siRNA targeting EZH2 and the negative scrambled sequence control were purchased from Ribobio (Guangzhou, China). PC9 Lenti-negative control (NC) and PC9 Lenti-HOTAIR cells were plated in six-well plates for 24 h and then transfected with specific siRNAs using Lipofectamine RNAi MAX in serum free medium, according to the manufacturer's protocol (Invitrogen, Grand Island, NY, USA). Cells were harvested 48 h after transfection at 70%-80% confluence. For lentiviral infection, HOTAIR overexpression or EZH2 shRNA lentiviruses (1 × 10^8^ transfection units TU/ml) and the vector control (1 × 10^8^ TU/ml) were purchased from GeneChem (Shanghai, China). The infected stable PC9 or PC9/AB2 cells were selected with 4 µg/mL puromycin dihydrochloride (Amresco) 72 h after lentiviral infection. The transfection efficiency was analyzed by RT-qPCR and western blot.

### CCK-8 assay

Approximately 3 × 10^3^ exponential growth phase cells per well were seeded into a 96-well plate and incubated overnight. Subsequently, the indicated concentration of gefitinib or other inhibitors was added for 48 h. Then, 20 μl of CCK-8 solution was added to each well and incubated for 1 h. Optical density was measured at 450 nm using a microplate reader to determine cell proliferation ability. All experiments were repeated four times independently. The IC50 was defined as the drug concentration needed for a 50% reduction in the absorbance, calculated based on cell survival curves. GraphPad Prism7.0 was used for the analysis.

### Western blotting

Western blotting was performed as previously described 20. Briefly, protein extracts were separated by 10% SDS-PAGE and transferred to polyvinylidene fluoride membranes (Millipore, USA). The membranes were incubated at 4 °C overnight with the following primary antibodies: anti-GAPDH (1:2000, Cell Signaling Technology); anti-p16 (1:1000, Abcam); anti-p21 (1:1000, Cell Signaling Technology); anti-CDK4 (1:1000, Cell Signaling Technology); anti-CDK6 (1:1000, Sigma); anti-CyclinD1 (1:1000, Cell Signaling Technology); anti-EZH2 (1:1000, Cell Signaling Technology); anti-LSD1 (1:1000, Cell Signaling Technology); anti-E2F1 (1:1000, Cell Signaling Technology); anti-H3K27 (1:1000, Cell Signaling Technology); anti-H3K4 (1:1000, Cell Signaling Technology); anti-Rb (1:1000, Cell Signaling Technology); anti-p-Rb ser-780 (1:1000, Cell Signaling Technology); and anti-p-Rb ser-795 (1:1000, Cell Signaling Technology). The membranes were then incubated with an anti-rabbit/mouse IgG secondary antibody (CST, USA). The blots were developed with ECL western blotting substrate (Pierce, USA).The bands on the membranes were detected using a Syngene G-Box and GeneSnap software (Syngene, Cambridge, UK).

### Real-time polymerase chain reaction (qRT-PCR)

Total RNA was isolated from cells using Trizol reagent (Invitrogen, CA, USA), according to the manufacturer's instructions. RNA was quantified using a UV spectrophotometer (Beckman Coulter, CA). Total RNA (2 μg) was reverse transcribed into complementary DNA (cDNA) using reverse transcriptase (Promega, Beijing, China). Gene amplification was achieved using qRT-PCR and Power SYBR Green PCR Master Mix (Applied Biosystems, Foster, CA, USA). Real-time PCR was carried out on an ABI 7500HT Fast Real-time PCR system (Applied Biosystems). GAPDH was used as an internal control.The relative expression of genes was calculated using the 2^-ΔΔCT^ method.

### Flow cytometry assay of cell cycle

Cells were stimulated with the indicated concentration of gefitinib for 48 h. Cells were then collected, resuspended in cold phosphate-buffered saline (PBS), and permeabilized in 70% ice-cold ethanol overnight. The cell suspension was mixed with 0.5 mg/mL propidium iodide and 50 mg/mL RNase A for 15 min in the dark using the PI/RNase Staining Buffer (BD Biosciences), according to the manufacturer's instructions. The cell cycle was analyzed using a FACSAria™ flow cytometer (BD Bioscience) and ModFit LT software (Verity Software House, ME, USA).

### 5-Ethynyl-2'-deoxyuridine (EdU) staining

After treating cells with the indicated concentration of gefitinib or inhibitors, the cells were incubated in 96-well plates for 24 h. Cells were then cultured with 50 μM EdU (RiboBio, Guangzhou, China) for 2 h followed by two washes with PBS and fixation with 4% paraformaldehyde. After penetration by 0.5% Triton X-100 and washing with PBS, the cells were dyed with Apollo (Red) and Hoechst 33342 (Blue) in the dark for 30 min. Stained cells were visualized by fluorescence microscopy.

### Chromatin immunoprecipitation (ChIP)

The ChIP was conducted according to the instructions of the Millipore Magna ChIP^TM^ A/G kit (Catalog # 17-10085, Millipore, USA). Briefly, cells were cross-linked with 1% formaldehyde for 10 min at room temperature. Subsequently, cells were treated with lysis buffer and sonicated for 30 min. Supernatants were treated with 5 μg of anti-H3K27me3 antibody (Cell Signaling Technology) for immunoprecipitation. Normal rabbit IgG acted as the control. The immunoprecipitated DNAs were identified by PCR.

### Immunohistochemistry analysis

The tissues for IHC staining were from in vivo experiments of our previous 20. Sections were deparaffinized with xylol and rehydrated through graded alcohol solutions (95, 70, and 50%). For antigen retrieval, sections were heated in 5 mM Tris-HCl buffer for 15 min at 100 °C. Endogenous peroxidase activity was blocked with 3% H_2_O_2_ and 5% bovine serum albumin (Beyotime Biotechnology, Shanghai, China). After a 10 min blocking step with normal goat serum, the tissues were incubated with anti-p16 or p21 antibody (Cell Signaling Technology, USA) at room temperature for 1 h. Sections were subsequently washed with PBS and incubated for 60 min with the horseradish peroxidase (HRP)-conjugated secondary antibody (Beyotime Biotechnology, Shanghai, China). Proteins were visualized in situ with 3,3-diaminobenzidine (DAB).

### Statistical analysis

SPSS 21.0 software (IBM, Chicago, IL) was used to perform statistical analysis in each experiment. Results were expressed as mean ± standard deviation (SD). The differences between the two groups were compared with the Student's *t*-test. A *p*-value <0.05 was considered statistically significant.

## Results

### Upregulation of HOTAIR enhances gefitinib resistance in NSCLC cells

**Figure 1:**
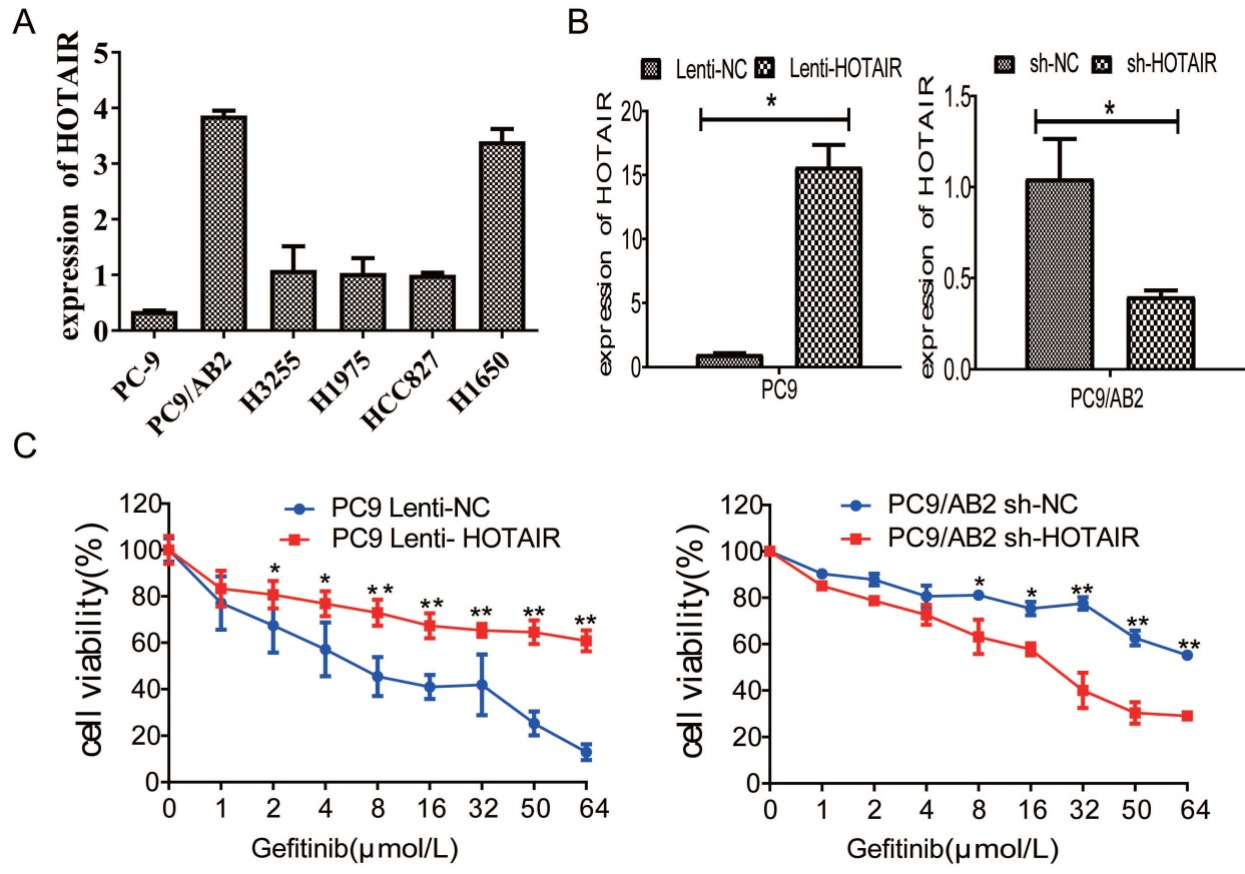
** The expression of HOTAIR is upregulated in gefitinib-resistant NSCLC cells.** (A) The expression of HOTAIR in six human lung cancer cell lines was detected by real-time qPCR. (B) The expression of HOTAIR was detected by real-time qPCR in PC9 cells stably expressing HOTAIR (PC9 lenti-HOTAIR) and PC9/AB2 cells stably expressing HOTAIR shRNA (PC9/AB2 sh-HOTAIR). *p < 0.05. (C) Cell viability was measured using a CCK-8 assay in PC9 cells stably expressing HOTAIR and PC9/AB2 cells stably expressing HOTAIR shRNA at different doses of gefitinib. *p < 0.05,**p < 0.01.

To determine whether HOTAIR plays a role in developing resistance to EGFR-TKIs in NSCLC, we measured HOTAIR expression in different NSCLC cells by qRT-PCR. HOTAIR was upregulated in gefitinib-resistant cell lines (PC9/AB2, H1650, and H1975) compared to gefitinib-sensitive cells (PC9, HCC827, and H3255). Gefitinib-resistant PC9/AB2 cells expressed the highest level of HOTAIR, while gefitinib-sensitive PC9 cells expressed the lowest HOTAIR level (Fig. 1A). To validate the role of HOTAIR in gefitinib resistance, we established the PC9-HOTAIR cell line, which stably overexpresses HOTAIR, and the AB2-shHOTAIR cell line, which stably represses HOTAIR expression, using lentivirus vectors. HOTAIR expression in the cell lines was verified by qRT-PCR (15.569 vs. 1; 0.400 vs. 1, *p* < 0.01, respectively, compared to NC groups) (Fig. 1B). Overexpression of HOTAIR enhanced gefitinib resistance in PC9 cells and the IC50 value of gefitinib in PC9-HOTAIR was significantly increased compared to the PC9-control cell line (108.6 μmol/L vs 17.8 μmol/L, *p* < 0.001). Knocking down of HOTAIR significantly reduced the survival of AB2 cells upon exposure to gefitinib. The IC50 for gefitinib in AB2-shHOTAIR and AB2-control cell lines were 19.45 μmol/L and 58.21 μmol/L, respectively (Fig. 1C). These results demonstrate that the upregulation of HOTAIR promotes gefitinib resistance in lung cancer cells and the silencing of HOTAIR sensitizes cells to gefitinib.

### HOTAIR overexpression promotes cell cycle progress and gefitinib resistance in lung cancer

**Figure 2:**
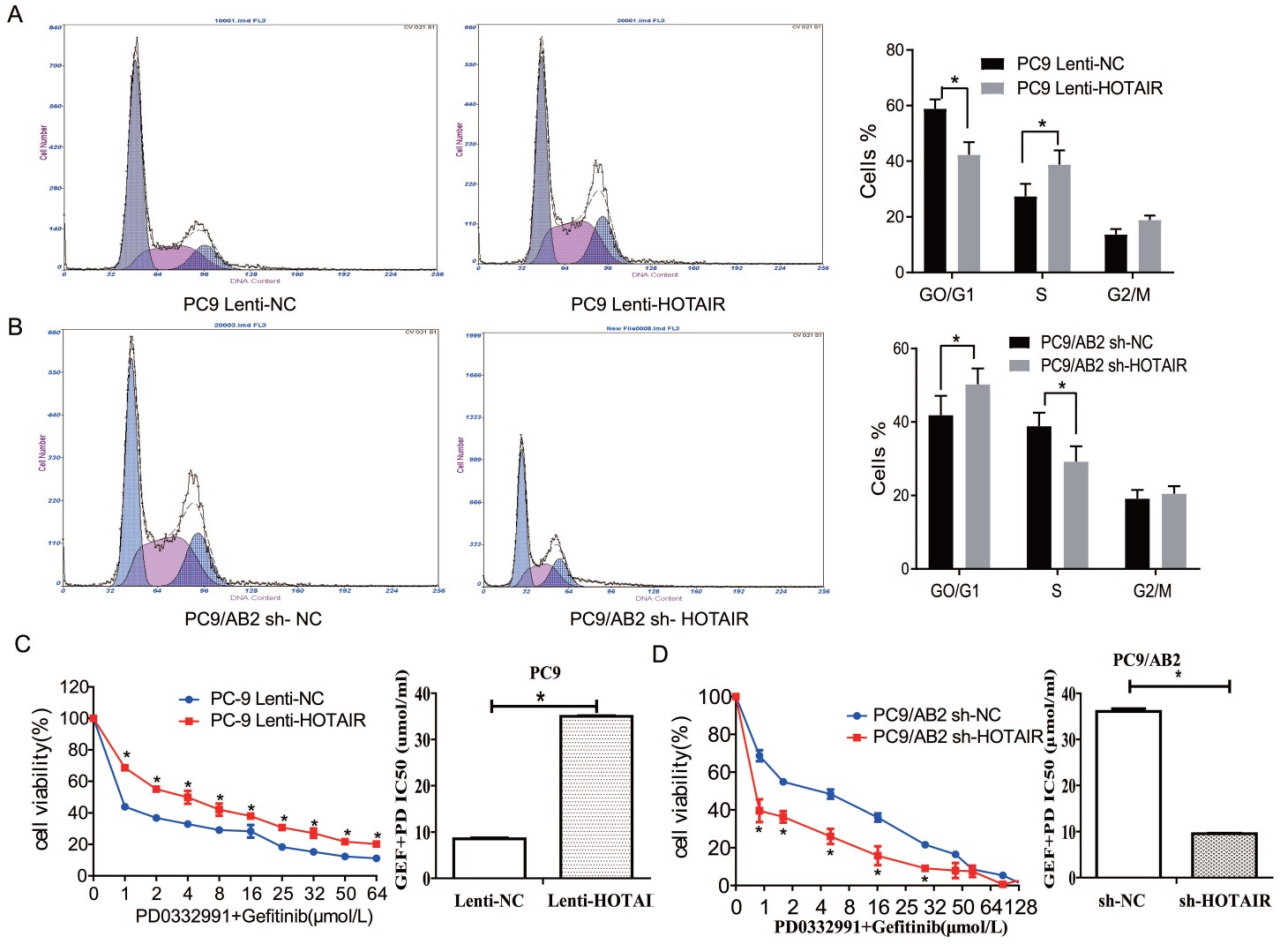
** Overexpression of HOTAIR promotes cell cycle progression in lung cancer.** (A-B) Cell cycle stages were analyzed by flow cytometry in PC9 lenti-HOTAIR and PC9/AB2 sh-HOTAIR cells to explore the effects of HOTAIR on the cell cycle. *p < 0.05. (C-D) Cell viability was measured using a CCK-8 assay in PC9 lenti-HOTAIR and PC9/AB2 sh-HOTAIR cells with different doses of gefitinib combined with PD 0332991. *p < 0.05.

Our previous report demonstrated that HOTAIR is a marker for abnormal cell cycle regulation in lung cancer 20. To investigate the effects of HOTAIR on the cell cycle and gefitinib sensitivity in NSCLC cells, cell cycle analyses were done using flow cytometry in PC9-HOTAIR, AB2-shHOTAIR and HCC827 cells. Compared with PC9-control cells, the percentage of PC9-HOTAIR cells in S phase was significantly increased (38.8% vs. 27.4%, *p* < 0.05) (Fig. 2A). The similar result was observed in HCC827 cells (Supplementary Fig. 1C-D).While the percentage of AB2-shHOTAIR cells in S phase was significantly decreased compared with AB2-control cells (29.2% vs. 38.9%, *p* < 0.05, Fig. 2B).

We previously demonstrated that PD 0332991, an inhibitor of cyclin-dependent kinase (CDK)4/6, could sensitize lung cancer cells to EGFR-TKI 22. To explore whether PD 0332991 interferes with HOTAIR effects on lung cancer cells, PC9-HOTAIR cells and AB2-shHOTAIR cells were treated with 5 μmol/L PD0332991 for 48 h. HOTAIR still promoted cell proliferation of PC9-HOTAIR cells in the presence of PD0332991 (IC50 12.36 μmol/L vs. 26.32 μmol/L) (Fig. 2C). The similar result was also observed in HCC827 cells (Supplementary Fig. 1E).When HOTAIR was silenced by shRNA, the survival of gefitinib-resistant PC9/AB2 sh-HOTAIR cells was reduced significantly compared to the control group (IC50 38.13 μmol/L vs. 12.32 μmol/L) (Fig. 2D) in the presence of PD 0332991. These results indicate that HOTAIR plays a specific role in regulating the cell cycle of NSCLC cells *in vitro*.

**Figure 3:**
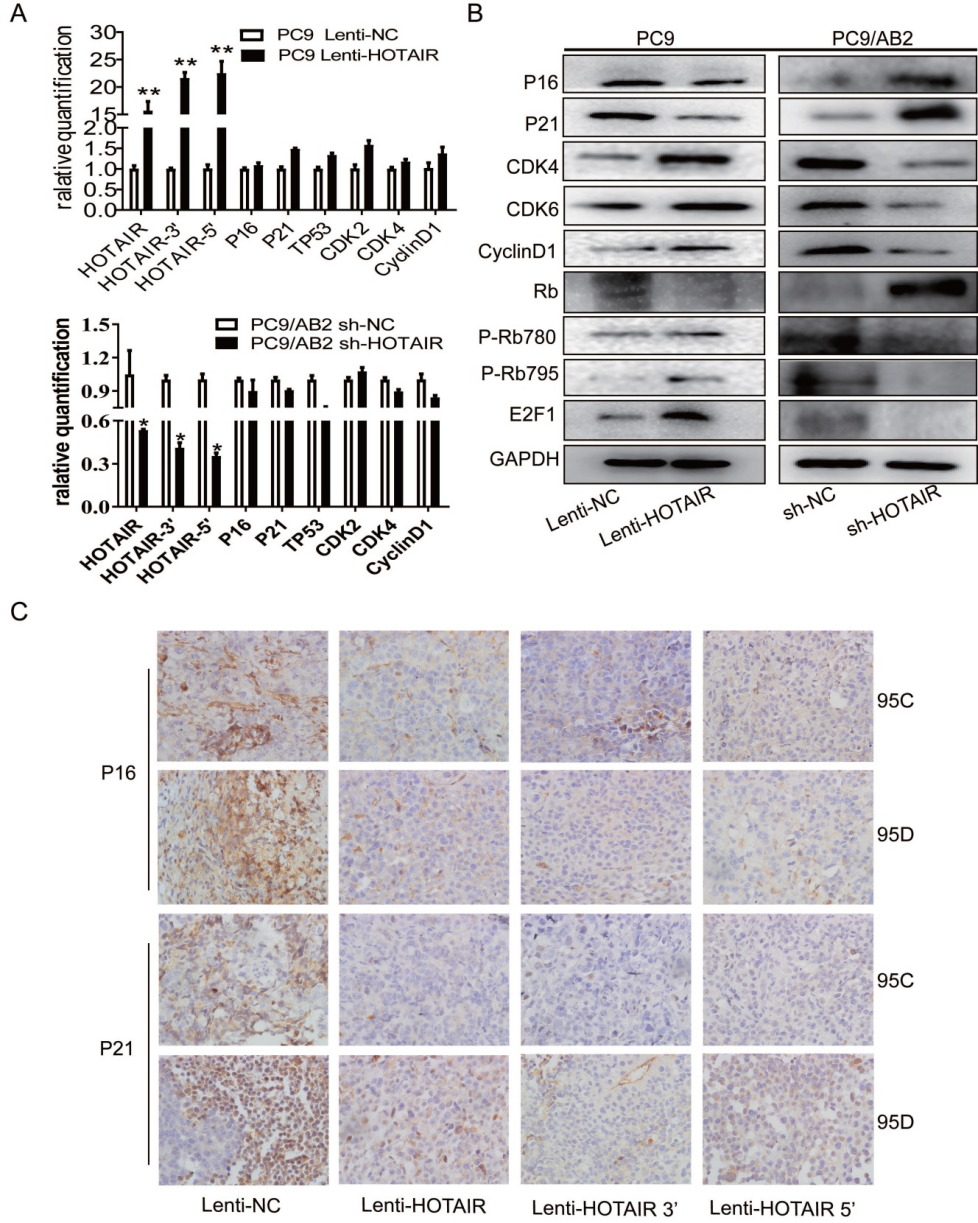
** HOTAIR suppresses p16 and p21 to accelerate cell cycle progression in NSCLC.** (A) HOTAIR, p16, p21, TP53, CDK2, CDK4, and CyclinD1 expression levels in PC9 lenti-HOTAIR and PC9/AB2 sh-HOTAIR cells were detected by real-time qPCR. *p < 0.05, **p < 0.01.(B) Cell cycle-related protein levels were assessed by western blot analysis. (C) Immunohistochemical staining was performed to detect p16 and p21 expression in animal tumor tissues.

To explore the regulatory effects of HOTAIR on cell cycle signaling, we measured mRNA levels of key molecules in the RB-E2F signaling pathway in PC9-HOTAIR and AB2-shHOTAIR cells. No significant changes in mRNA levels were observed in cell cycle genes (p16, p21, CDK2, CDK4, and CyclinD1) in either the PC9-HOTAIR cells or the AB2-shHOTAIR cells (Fig. 3A). Interestingly, HOTAIR dramatically suppressed p16, p21, and RB protein expression, but enhanced the protein levels of CDK4, CDK6, E2F1, and CyclinD1 (Fig. 3B, and Supplementary Fig. 2A). In our previous study, we established lung cancer xenograft mouse models using 95C and 95D cells transfected with Lenti-NC, Lenti-HOTAIR, Lenti-HOTAIR3', and Lenti-HOTAIR5. P-Rb (S795), CDK4, and CDK6 were highly expressed in HOTAIR, HOTAIR3', and HOTAIR5' over-expressed groups compared with the controls, whereas Rb expression was increased in the orthotopic NSCLC model transfected with Lenti-shHOTAIR 21. Here, immunohistochemical stains was employed to detect the expression of p16 and p21 proteins on paraffin sections made from the lung cancer xenograft mouse models in our previous study 20. Our results showed that overexpression of HOTAIR reduced the expression of p16 and p21 (Fig. 3C). Together, our results indicate that HOTAIR promotes cell cycle progression and gefitinib resistance in NSCLC cells.

### HOTAIR regulates cell cycle progression through epigenetic regulation of EZH2/H3K27

**Figure 4:**
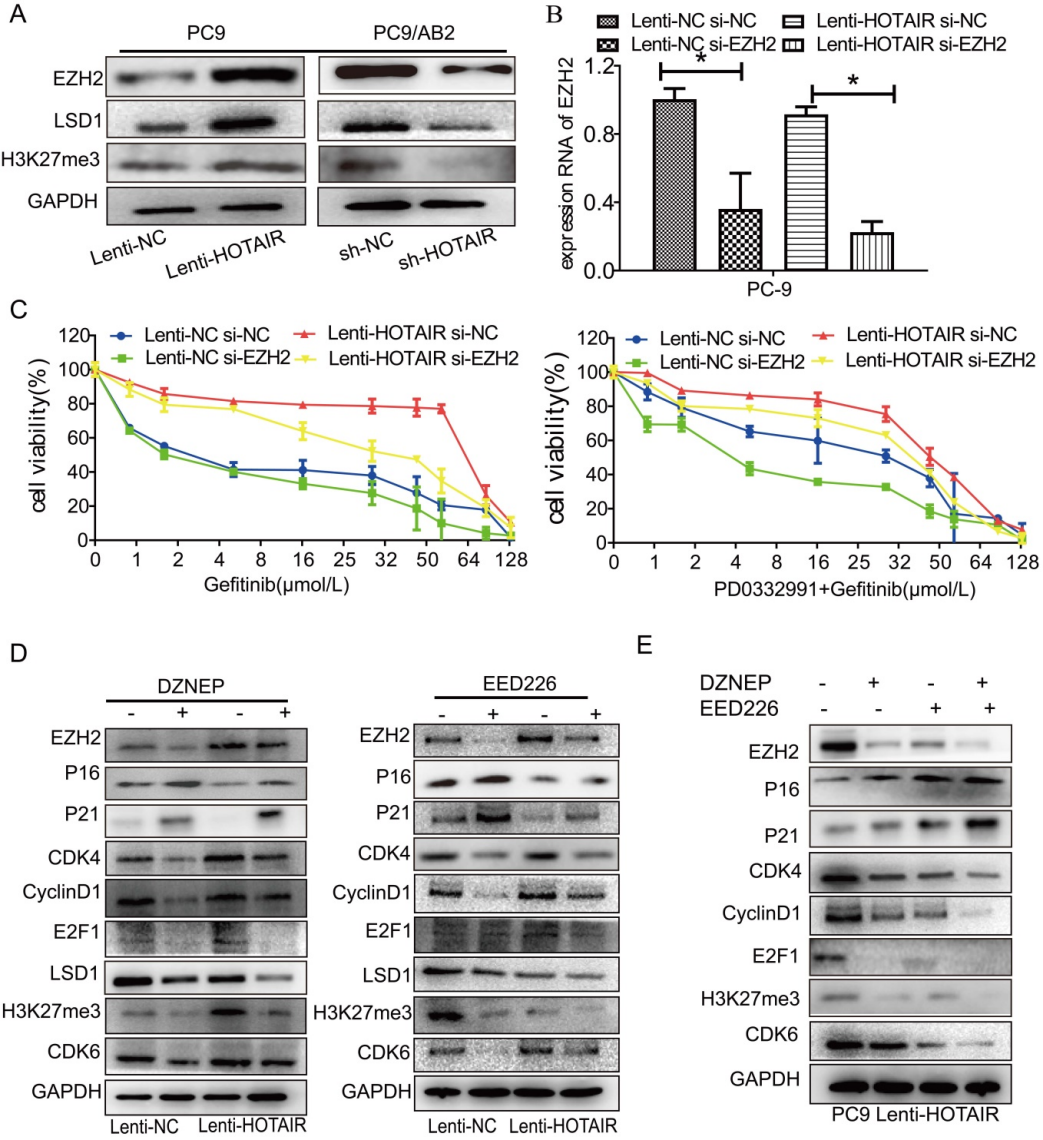
** HOTAIR regulates drug resistance through EZH2/H3K27 epigenetic regulation of the cell cycle in lung cancer cells.** (A) Western blot analysis was used to examine the influence of HOTAIR on the expression of EZH2, LSD1, H3K27me3 proteins in both PC9 lenti-HOTAIR and PC9/AB2 sh-HOTAIR cells. (B) EZH2 expression levels in PC9 lenti-HOTAIR were detected using real-time qPCR after transfection with EZH2 siRNA or NC for 48 h. *p < 0.05. (C) Cell viability was detected using a CCK-8 assay in PC9 lenti-HOTAIR and PC9/AB2 sh-HOTAIR cells transfected with EZH2 siRNA or NC for 48 h after treatment with different doses of gefitinib alone or combined with PD 0332991. (D) Western blot analysis was used to detect the expression of EZH2, p16, p21, H3K27me3, and other cell cycle related proteins in PC9/lenti-NC and PC9/lenti-HOTAIR cells treated with DZNEP or EED226. (E) The expression levels of EZH2, p16, p21, H3K27me3, and other cell cycle related proteins were analyzed in PC9/lenti-HOTAIR cells after combining treatment of DZNEP and EED226, or with either DZNEP or EED226 given alone

Hyperactivity of HOTAIR promotes malignancy through interaction with the polycomb repressive complex 2 (PRC2) complex. The PRC2 complex is composed of four conserved core components (EZH1/2, SUZ12, EED, and RbBP4) and several other proteins. Enhancer of zeste homolog 2 (EZH2), a core molecule of the PRC2 complex, catalyzes monomethylation, dimethylation, and trimethylation of lysine 27 on histone H3 (H3K27) 23,24. To explore the possible mechanisms of HOTAIR-induced resistance to gefitinib, the PRC2 complex was analyzed by western blotting. As shown in Fig. 4A, overexpression of HOTAIR in PC9-HOTAIR cells led to the increased expression of H3K27, EZH2, and LSD1, while repression of HOTAIR expression led to the decreased expression of these three proteins. Furthermore, when EZH2 was silenced by siRNA, the CCK8 assay revealed that cells were sensitized to gefitinib (Fig. 4B and 4C). The IC50 values for gefitinib in PC9 cells transfected with HOTAIR. HOTAIR plus siRNA-EZH2, siRNA-EZH2, and the negative control were 194.6 μmol/L, 95.93 μmol/L, 30.33 μmol/L, and 41.87 μmol/L, respectively. When gefitinib was combined with PD0332991, the IC50 values for gefitinib in the same groups were 132.1 μmol/L, 92.31 μmol/L, 40.16 μmol/L, and 74.69 μmol/L, respectively (Fig. 4C).

Two small molecule inhibitors (DZNEP, a potent EZH2 inhibitor, and EED226, a PRC2 inhibitor) were used to verify the roles of EZH2 and PRC2 in HOTAIR-mediated gefitinib resistance in PC9 cells. As shown in Fig. 4D and Supplementary Fig. 2B, EZH2 protein expression was decreased after DZNEP and EED226 treatments in both PC9 and HCC827 cells. Compared to the untreated cells, the expression levels of p16 and p21 were significantly increased by DZNEP or EED226 in the PC9 and HCC827 cells. Furthermore, compared to the untreated cells, the expression levels of CDK4, cyclinD1, E2F1, and LSD1 were significantly decreased in the PC9-control and PC9-HOTAIR groups after DZNEP or EED226 treatments. Interestingly, compared to the untreated cells, the expression of CDK6 protein was significantly increased in the NC group and decreased in the lenti-HOTAIR group after treatment with DZNEP or EED226. The similar result was also observed in HCC827 cells (Supplementary Fig. 2B). Since EZH2 catalyzes H3K27 methylation 23, we also examined H3K27 methylation in this study. Compared to untreated cells, the methylation protein of H3K27 was also decreased in both groups with DZNep or EED226 treatment (Fig. 4D and Supplementary Fig. 2B). Similar results are shown in PC9-HOTAIR cells with DZNEP and EED226 combination treatment (Fig. 4E). Altogether, our data indicated that HOTAIR regulates cell cycle progression through epigenetic regulation of EZH2/H3K27 in non-small cell lung cancer cells.

### HOTAIR/PRC2 suppresses p16 and p21 and accelerates gefitinib resistance in NSCLC cells in vitro and in vivo

**Figure 5:**
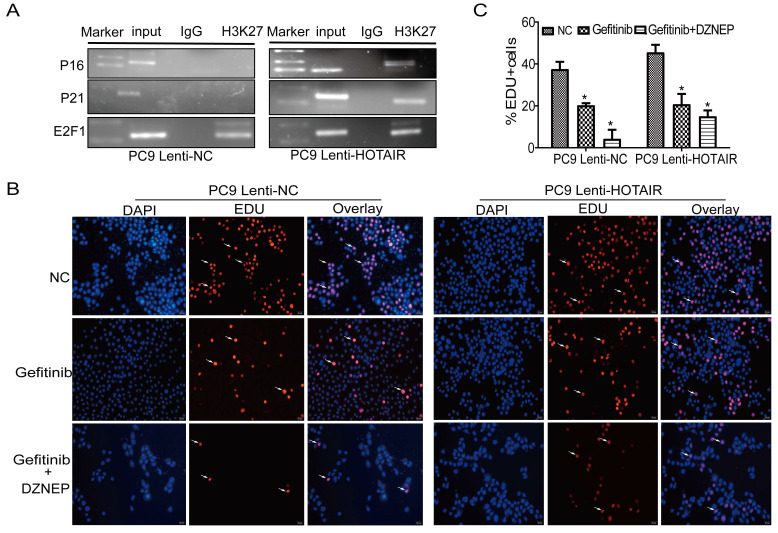
** HOTAIR increased H3K27me3 recruitment to the promoter of p16 and p21.** (A) ChIP assay was used to detect the enrichment of p16 and p21 using anti-H3K27 antibody. (B-C) Cell proliferation activity was examined using an EdU assay in PC9 cells treated with gefitinib alone or in combination with DZNEP. *p < 0.05.

To further explore the mechanism by which HOTAIR regulates the cell cycle and gefitinib resistance, we carried out a ChIP assay to determine if HOTAIR acts together with PRC2 in regulating p16 and p21 expression in PC9 cells. As shown in Fig. 5A, using PC9/lenti-NC and lenti-HOTAIR cells, the anti-H3K27 antibody was used to immunoprecipitate chromatin containing DNA fragments that have the promoter regions of p16, p21, and E2F1 genes. In the ChIP-PCR experiments, the PCR bands of p16 and p21 promoters were shown in PC9-HOTAIR cells, but not in PC9/lenti-NC cells. The PCR bands of the E2F1 promoter existed in both PC9/lenti-NC and lent-HOTAIR cells as a positive control. These data demonstrate that the overexpression of HOTAIR increases H3K27me3 recruitment to the promoters of p16 and p21. Furthermore, the EdU staining assay demonstrates that DZNEP eliminates the promotion of cell proliferation by overexpression of HOTAIR in PC9/lenti-HOTAIR cells. The positive rates of EdU staining in the Gefitinib + DZNEP, Gefitinib, and NC groups were 15.0%, 20.7%, and 45.5%, while cell proliferation was significantly different in PC9-control cells with different treatments (4.3%, 20.3%, and 37.5%, respectively) (Figs. 5B-C). These data confirm that EZH2 is involved in HOTAIR-induced cell cycle progression in lung cancer cells. HOTAIR cooperates with EZH2 to silence p16 and p21 expression, mediated by H3K27me3 in the p16 and p21 promoter region.

The above results in Fig 3C showed that overexpression of HOTAIR leads to an decrease of p16 and p21 protein levels *in vivo*.Altogether, our results indicate that HOTAIR silences p16 and p21 and accelerates cell cycle progression through post-transcriptional regulation in NSCLC cells. HOTAIR/PRC2 suppresses p16 and p21 and accelerates gefitinib resistance in NSCLC cells *in vitro* and *in vivo*.

## Discussion

Gefitinib, a TKI targeting EGFR, has been successfully used for first-line treatment of NSCLC patients with sensitive EGFR mutations. Unfortunately, many patients treated with gefitinib inevitably develop resistance to the drug after 6-12 months of treatment, leading to limited clinical application and treatment failure. Therefore, a better understanding of the mechanism of EGFR-TKI resistance is crucial. LncRNAs act as regulators of multiple cellular processes, including the cell cycle, survival, apoptosis, and metastasis 25. One of the most studied lncRNAs is HOTAIR 25. Several lines of evidence have recently pointed to the functional role of dysregulated HOTAIR in the initiation and progression of chemotherapy resistance in several solid tumors, where HOTAIR expression is upregulated and associated with poor prognosis in NSCLC patients 26-29. For example, the downregulation of HOTAIR sensitizes breast cancer to trastuzumab 19. HOTAIR contributes to Polyphyllin I-inhibited growth of castration-resistant prostate cancer cells by regulating DNMT1 and EZH2 in prostate cancer 30. HOTAIR inhibits cisplatin resistance of gastric cancer cells through inhibition of the PI3K/Akt and Wnt/β-catenin signaling pathways 31. In this study, we show that HOTAIR is highly expressed in gefitinib-resistant cell lines, such as PC90/AB2, H1650, H1975 cells, compared with sensitive cell lines, such as PC9 and HCC827 cells. To explore the role of HOTAIR in gefitinib resistance, we upregulated HOTAIR expression in gefitinib-sensitive lung cancer PC9 cells and established HOTAIR-overexpressing cell lines. Meanwhile, we performed loss-of-function studies by knocking down HOTAIR in gefitinib-resistant lung cancer PC9/AB2 cells. Similar to the previous studies, HOTAIR overexpression led to gefitinib resistance in lung cancer cells. In addition, high expression of HOTAIR promoted cell cycle progression in gefitinib-resistant lung cancer cells. Our results suggest that HOTAIR contributes to gefitinib resistance through promoting cell cycle in NSCLC.

The cell cycle is tightly regulated by a series of events that govern cell replication and division. Cell cycle dysregulation is a common feature of human cancers. p16 and p21 are two critical proteins in the cell cycle and their dysregulation has been tied to different malignant cancers 32,33. p16 is an inhibitor of CDKs and prohibits cell cycle progression from the G1 phase to the S phase, mediated by pRB-E2F repressor complexes 34, whereas p21 arrests the cell cycle progression in G1/S and G2/M transitions by inhibiting the activity of cyclin-CDK2, -CDK1, and -CDK4/6 complexes 35. We previously reported that HOTAIR influences cell cycle regulation and is a marker of cell cycle dysregulation in lung cancer. In our current study, overexpression of HOTAIR induces gefitinib resistance in lung cancer cells through inhibition of p16 and p21 gene expression, where HOTAIR may silence the p16 and p21 genes thereby promoting the expression of CDK4, CDK6, E2F1, and CyclinD1 to accelerate cell cycle progress in drug-resistant lung cancer cells. This mechanism is supported by our finding that overexpression of HOTAIR enhances the expression levels of CDK4, CDK6, E2F1, and CyclinD1 proteins.

HOTAIR reprograms chromatin by genome-wide re-targeting of PRC2 to an occupancy pattern, leading to altered histone H3K27 methylation 23,24. EZH2, the catalytic subunit of PRC2, directly binds to HOTAIR in different models. HOTAIR promotes glioblastoma cell cycle progression in an EZH2-dependent manner. In the present study, HOTAIR overexpression led to increased H3K27 methylation. To explore how EZH2 is involved in HOTAIR-mediated repression of p16 and p21, DZNep and EED226 were used. DZNep is an EZH2 inhibitor that induces the degradation of PRC2 by impairing SAH (S-adenosyl-l-homocysteine). EED226 is a small molecule inhibitor that blocks the H3K27me3-binding pocket of EED. Our results showed that p16 and p21 were significantly increased after DZNEP or EED226 treatment, whereas CDK4, cyclinD1, E2F1, LSD1, and H3K27 methylation significantly decreased after DZNEP or EED226 treatment in cells without overexpression of HOTAIR. These results indicate that the overexpression of HOTAIR increases H3K27me3 recruitment to the promoter of p16 and p21, implying that HOTAIR cooperates with EZH2 to silence p16 and p21 expression mediated by H3K27me3 in their promoter regions.

In conclusion, our study revealed that HOTAIR contributes to gefitinib resistance. Overexpression of HOTAIR promotes cell cycle progression by silencing p16 and p21 cooperating with EZH2 in human drug-resistant lung cancer cells. Our results demonstrate that HOTAIR can act as a predictive biomarker of acquired gefitinib resistance and may be a promising target for therapeutic intervention in patients with gefitinib resistance.

## Supplementary Material

Supplementary figures.

## Abbreviations

HOTAIR: HOX Transcript Antisense RNA
lncRNA: Long noncoding RNA
EZH2: Enhancer Of Zeste 2 Polycomb Repressive Complex 2 Subunit
NSCLC: non-small cell lung cancer
qRT-PCR: quantitative real-time polymerase chain reaction
ChIP: Chromatin immunoprecipitation
TKI: Tyrosine Kinase Inhibitor
