# Supplementary material for: HOTAIR promotes gefitinib resistance through modification of EZH2 and silencing p16 and p21 in non-small cell lung cancer
Source: J Cancer. 2021 Jul 25;12(18):5562–72. doi: 10.7150/jca.56093 (PMC8364642; doi:10.7150/jca.56093)
Supplement: Supplementary file 1 — Supplementary figures. [file jcav12p5562s1.pdf]

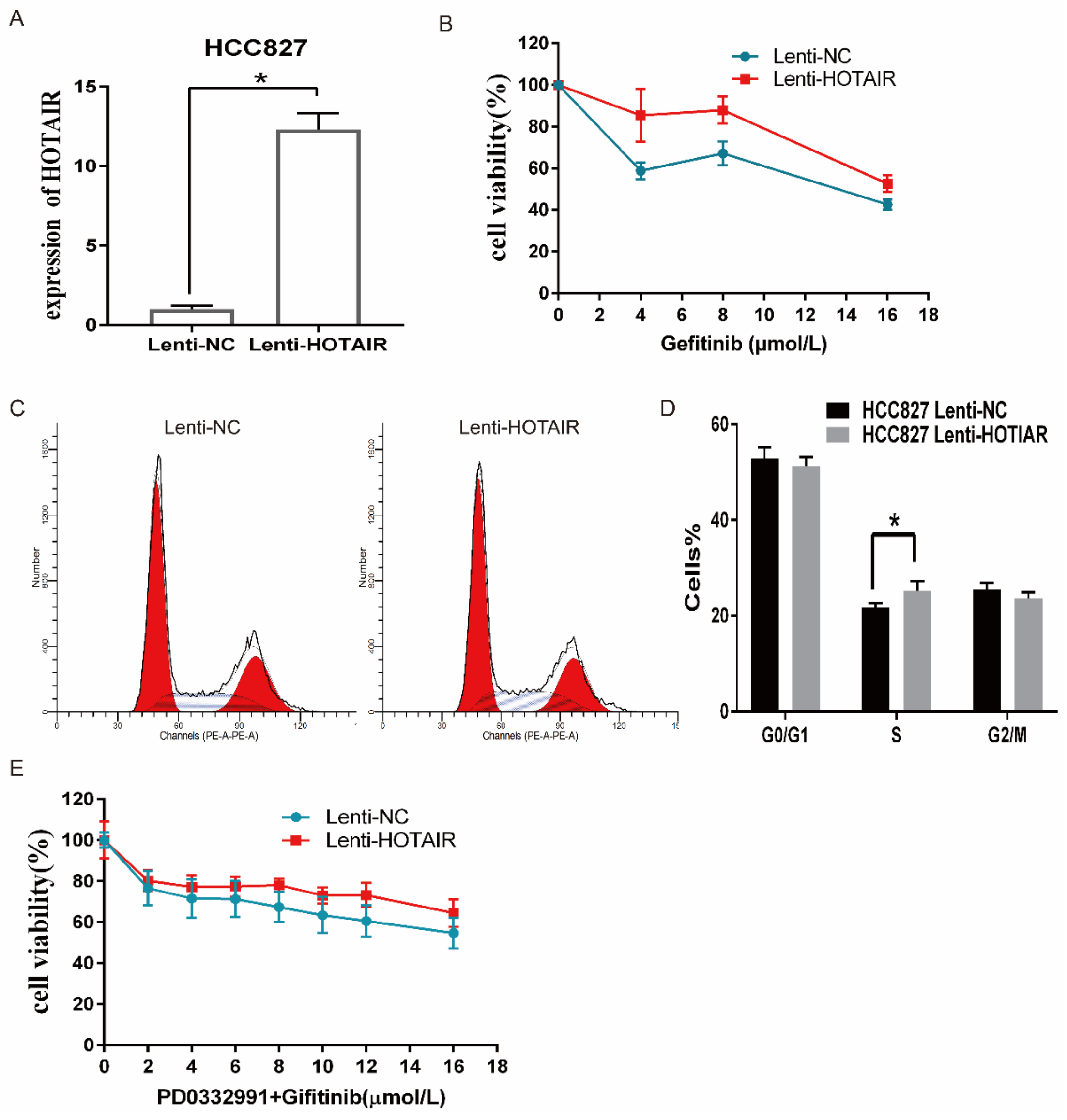

suppl Figure 1. The effort of HOTAIR on cell growth of HCC827 cells. (A)The expression of HOTAIR was detected by real-time qPCR in HCC827 cells stably expressing HOTAIR or NC . (B) Cell viability was measured using a CCK-8 assay in HCC827 cells stably expressing HOTAIR at different doses of gefitinib.(C-D) Cell cycle stages were analyzed by flow cytometry in HCC827 lenti-HOTAIR and lenti-NC. *p < 0.05.(E). Cell viability was measured using a CCK-8 assay in HCC827 lenti-HOTAIR and lenti-NC. with different doses of gefitinib combined with PD 0332991.

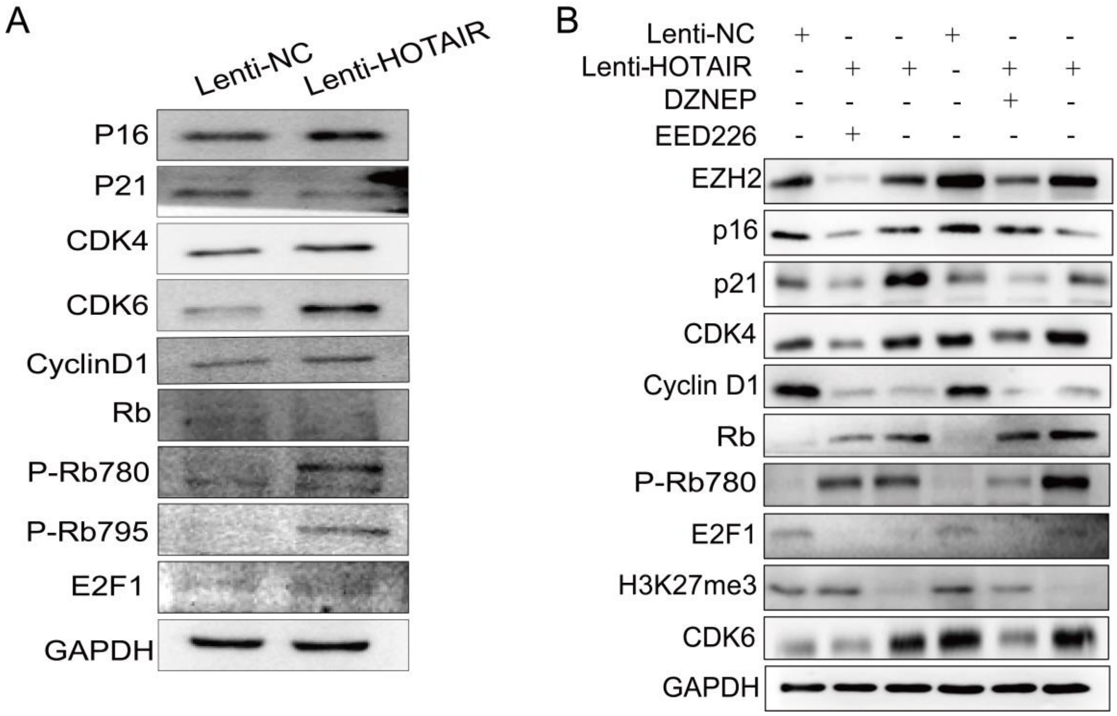

suppl Figure 2. HOTAIR suppresses p16 and p21 to accelerate cell cycle progression in HCC827 cells. (A) Cell cycle-related protein levels were assessed by western blot analysis in HCC827 cells stably expressing HOTAIR or NC . (B) Western blot analysis was used to detect the expression of EZH2, p16, p21, H3K27me3, and other cell cycle related proteins in in HCC827 cells stably expressing HOTAIR or NC treated with DZNEP or EED226
